# Misinformation and Profitability of Hepatitis B Virus Claims on Instagram: Formative Cross-Sectional Study

**DOI:** 10.2196/71584

**Published:** 2026-03-02

**Authors:** Zachary C Warner, Cindy A Turner, Rodrigo Alvarez, Juan F Gallegos-Orozco, Ashley Green, Echo L Warner

**Affiliations:** 1 Division of Gastroenterology, Hepatology and Nutrition School of Medicine University of Utah Salt Lake City, UT United States; 2 Huntsman Cancer Institute Salt Lake City United States; 3 Department of Internal Medicine University of Arizona Tucson, AZ United States; 4 University of Arizona Cancer Center Tucson United States; 5 College of Nursing University of Utah Salt Lake City, UT United States

**Keywords:** misinformation, hepatitis B, Instagram, social media, vaccine

## Abstract

**Background:**

The internet is increasingly used to find health information, which often contains misinformation. Instagram is a likely source of health information online for many adults worldwide, given that there are more than 2 billion worldwide users. To date, no studies have documented the characteristics of hepatitis B virus (HBV) claims, information accuracy, engagement with, and profitability of HBV information on Instagram.

**Objective:**

We aimed to document the characteristics, accuracy, engagement, and profitability of HBV misinformation on Instagram.

**Methods:**

In this cross-sectional formative study, 2 research members searched for publicly available Instagram posts using the terms “hepatitis b” and “hep b” and manually extracted data from the most popular posts and user profiles for each term from December 2021 to January 2022 at varying times of the day and days of the week. We applied an existing and validated health misinformation codebook, adapted for this topic, to 103 posts for 58 variables, including post characteristics, types of HBV claims (eg, treatment, prevention, and cure), accuracy of information (misinformation vs accurate, coded by hepatology clinicians), engagement (number of likes), and profitability (yes or no). We calculated descriptive statistics and applied chi-square, Fisher exact, and *z* tests to compare posts with certain characteristics, claims, and engagement by accuracy and profitability in Stata (version 18.0) with significance set at an α of .05.

**Results:**

Of the full sample, most posts had accurate (79/103, 76.7%) versus inaccurate (24/103, 23.3%) information about HBV. Among posts with claims about HBV treatment (18/103, 17.5%), there were more posts that had misinformation than accurate posts (55.6% vs 44.4%; *χ*²_1_=12.7; *P*<.001). Similarly, there were higher proportions of posts with misinformation compared to posts with accurate information about cures (n=12, 75% vs 25%; Fisher *P*<.001), natural remedies (n=13, 92.3% vs 7.7%; Fisher *P*<.001), symptoms (n=15, 60% vs 40%; *χ*²_1_=13.2; *P*<.001), and censorship conspiracies (n=9, 66.7% vs 33.3%; Fisher *P*=.005) related to HBV. Compared to posts with accurate information, posts with misinformation had more likes on average (mean 1459.2, SD 1458.8-1459.6 vs mean 941.8, SD 941.6-942.0; *z*=−517.4; *P*<.001). Significantly more posts with misinformation were for profit (39.5% vs 13.8%; *χ*²_1_=8.8; *P*=.003) than accurate posts.

**Conclusions:**

HBV misinformation had more engagement than accurate information on Instagram and was more likely to be for-profit than accurate information. HBV misinformation may spread more easily than accurate information, meaning people searching for HBV on Instagram may encounter false, profit-driven claims that could affect health behaviors. Our focus on visual social media misinformation is innovative, as is our use of Instagram, an understudied platform. More research is needed to estimate the prevalence of HBV misinformation and its influence on health beliefs, behaviors, and outcomes. Improving media literacy may help reduce the influence of HBV misinformation online.

## Introduction

The use of social media to seek and share health information has dramatically increased in the past few decades and was exacerbated by the growth of contemporary medical mistrust during the COVID-19 pandemic [1,2]. While social media improves access to health information and social support, health misinformation is widespread on these platforms [3-6]. Given the growing use of social media for health information seeking, public calls have been made for hepatologists to “lead and contribute” to online conversations to promote science-based information about liver disease on social media [7]. However, little research has been conducted to characterize the content and engagement of the public with online hepatology information, and this research is needed to guide clinicians and researchers on how to intervene and promote high-quality information online to modern health consumers.

Health misinformation has been defined as “any health-related claim of fact that is false based on current scientific consensus” [8]. Misinformation has been shown to disseminate faster than accurate information on social media [9], and health-related misinformation can have detrimental effects on patients, such as the promotion of unproven and ineffective medical treatments [10,11]. Exposure to misinformation on social media in other health contexts (eg, cancer) is associated with elevated patient distress, confusion about medical decision-making, and harm to interpersonal relationships [4,12,13]. Exaggerated beliefs that certain foods or food components can cure or cause disease, or have special health benefits, originate from the concept of “food faddism” [14,15]. Health misinformation often promotes food faddism through extreme dietary shifts, use of potentially harmful supplements, and opting for unproven alternative treatments, and these behaviors are known to contribute to treatment nonadherence and to increase mortality up to 5-fold for some diseases (eg, curable cancers) [11,16]. These patterns of misinformation suggest a pathway by which exposure to online misinformation about health has an eventual impact of patient health decisions and health outcomes. To establish a foundation for this novel research area, we studied hepatitis B misinformation on Instagram, a popular social media website that comprises visual and textual content.

We focused on hepatitis B because of its high worldwide prevalence and chronic nature. It is estimated that worldwide as many as 291 million people may be living with the hepatitis B virus (HBV) [17]. While most individuals who are infected with HBV are asymptomatic, up to 15% to 40% will develop chronic disease [18]. Chronic HBV infection can cause chronic hepatitis, cirrhosis, and hepatocellular carcinoma. Hepatocellular carcinoma is the fourth leading cancer-related cause of death worldwide, with 68% of cases occurring in sub-Saharan Africa [18-20]. While there is no cure for hepatitis B, treatment of the virus can reduce both morbidity and cancer risk and requires lifelong management [21]. The high prevalence and potential seriousness of HBV sequelae suggest that hepatitis B is a commonly discussed topic on the internet, but this has not been previously documented.

Furthermore, social media are a common source of social connection for patients with chronic, life-threatening, and stigmatized diseases such as cancer and HIV [22-24] and potentially HBV. Patients with chronic HBV infections experience clinically significant levels of anxiety, depression, and negative psychosocial (eg, incarceration, homelessness, and drug use) and community (eg, stigma)–level outcomes [25,26]. Social exclusion and the risk of psychological sequelae of a chronic HBV infection may increase patients’ proclivity for online misinformation when they lack support from offline social networks [27]. Thus, patients with HBV are likely to turn to the internet to find information and support that they may otherwise not have access to offline. This pattern of simultaneous support and information seeking exists for patients with other diseases that have similar trajectories, such as cancer and HIV or AIDS [28].

Therefore, in this study, we aimed to document the characteristics and prevalence of HBV misinformation in Instagram posts, factors that may influence the impact these posts have on viewers, and engagement with HBV misinformation on Instagram. Instagram was the third most popular social media platform in the United States at the time of the study and has a wide global viewership estimated to be between 1.4 and 1.6 billion active users [29]. Instagram was selected for this study, given the paucity of research on visual and audio health misinformation, and Instagram is highly visual. We applied a deductive content analysis to classify the features of hepatitis B claims on Instagram using common search terms. This work was informed by a conceptual framework of online misinformation exposure, originally developed in an oncology setting [13]. Most studies of social media misinformation focus on vaccines, smoking and vaping products, or illicit drugs, with only 10% of studies about communicable diseases [30]. To our knowledge, this is the first study to evaluate the characteristics, prevalence, and reach of HBV misinformation and its association with profitability on social media.

## Methods

### Ethical Considerations

This study used publicly available social media data, which were considered exempt by the University of Arizona institutional review board. As no participants were recruited, no informed consent nor compensation was provided. We report analyses in aggregate, and all data are anonymized to eliminate the potential for reidentification of social media posts. No individual participants or users are identifiable in the paper or supplementary material. We followed the STROBE (Strengthening the Reporting of Observational Studies in Epidemiology) guidelines for reporting cross-sectional studies (Multimedia Appendix 1) [31].

### Conceptual Framework

To situate this work within existing online misinformation research in other domains, we applied the Online Cancer Nutrition Misinformation (OnC-M) framework, a classification tool that identifies categories of misinformation, effect modifiers, and potential impacts of misinformation on patient behavior, decision-making, and health outcomes [13]. The OnC-M framework includes a codebook and definitions of health misinformation claims developed through a series of studies that provide important context for generating hypotheses about health misinformation online [13,32-37].

### Instagram Platform

Instagram is a visual social media platform centered on content sharing through images and videos. Users share content, which can then be viewed and engaged with through likes and comments from their followers. Instagram is widely used in the United States, more commonly by female individuals than male individuals [29]. Our approach to collecting, coding, and analyzing data about hepatitis B–related claims on Instagram was informed by our prior studies of online health misinformation on Facebook, Instagram, Pinterest, and Twitter [13,35-37]. We adopted an existing codebook that was used in these prior studies to classify misinformation, which we modified to include hepatitis B content claims and began data collection.

### Data Collection

In December 2021, we searched Instagram using key search terms “hepatitis b” and “hep b.” Search terms were intended to represent lay language used by social media users and were selected through consensus among the research team and community engagement feedback. To describe the most viewed content on Instagram, we collected the top 55 posts populated for each term. This procedure is standard, given that algorithmically the most popular and engaging posts (ie, most viewed and liked) are more likely to populate first [38]. Selected posts were captured with a screenshot, and the profiles of users who posted the content were subsequently coded for descriptive characteristics (eg, number of followers). Screenshots were taken to enhance data quality and completeness. We aimed for a sample of 100 posts, with oversampling each term to account for duplication; duplicates were removed, resulting in a final sample of 103 posts. Each row in the dataset represented a unique post, which was the unit of analysis. On completing data collection, the coding for each post was quality checked by ZCW and ELW (eg, live links, data completeness, and duplication).

### Content Analysis

After finalizing the sample, each record was coded for 58 variables, which were chosen based on prior research [13,35-37], including characteristics of Instagram posts about HBV (graphics, creator expertise, creator status, target audience, world region, disclaimers, and mention of COVID-19), and content variables representing HBV-related claims (activism, treatment, natural treatment or cures, anecdotal experiences, prevention, cures, symptoms, censorship, and other). For each post, descriptive characteristics about the user and the post were manually coded from the Instagram platform and entered as variables into Microsoft Excel. We coded for 3 additional validity indicator variables that are known modifiers from the OnC-M framework, which in this study related to the quality of or truthfulness of HBV and nutrition-related claims. These 3 variables represented the inclusion of academic or government citations (yes or no), personal anecdotal experiences (yes or no), and the use of disclaimers to contextualize claims about hepatitis B (yes or no). Finally, given the previously discussed financial incentives for promoting unproven cures and treatments for diseases on social media, we coded an additional for-profit code to represent posts that were explicitly advertising or selling a product that claimed to treat or cure HBV. Variables were coded according to both the screenshots of posts and the poster’s Instagram user profile. Coding of the posts occurred through 2 cycles to establish reliability. Following standards for evaluating the accuracy of online health information, posts were evaluated for accuracy based on expert knowledge, including hepatology clinicians with technical and medical expertise, to identify the presence or absence of accurate information based on current standards of care, which was coded as accurate (score=0) versus misinformation (score=1). Examples of misinformation included false claims of HBV cures (“My herbal remedies can cure any kind of sickness and diseases...”) and HBV-related government conspiracies (“...expose the malfeasance, the coverups and the denials that run our health system”). In the first cycle, 10% of posts were inductively coded by 2 authors (ZCW and AG) to identify HBV-related claims. Areas of discrepant coding were reviewed, and decisions about new codes, refined definitions, and irrelevant content were made through coder consensus, with the input of a tiebreaker (ELW). The codebook was then applied to all the remaining posts. There were no missing data for which to account.

### Statistical Analysis

We summarized each variable to describe the characteristics of Instagram posts about HBV and types of HBV claims. While this was a cross-sectional study with primarily descriptive aims, we performed limited secondary inferential analyses to examine selected associations for the purpose of hypothesis generation, a common goal of formative research [39,40]. We compared the proportion of claims associated with misinformation to the proportion of claims without misinformation using *z* tests. Finally, we evaluated the associations of claims with validity indicators (citations, anecdotes, and disclaimers) and profitability using chi-square and Fisher exact tests.

## Results

### Characteristics of Instagram Posts About HBV

Most HBV posts contained a mix of images and text (38/103, 36.9%) or an image alone (n=31, 30.1%; Table 1). A minority group of content creators claimed to have some kind of health expertise either in the post or on their Instagram profile (n=38, 36.9%). Similarly, a minority group of content creators claimed to be for-profit (n=38, 36.9%), meaning the majority claimed to be from not-for-profit sources (n=65, 63.1%). Most posts (n=52, 50.5%) did not specify a particular audience, but of those that did, the majority were targeting patients and caregivers of patients with HBV (n=32, 31.1%). While HBV infection is the most prevalent in sub-Saharan Africa, most HBV posts (n=40, 38.8%) in our sample originated from the Americas, followed by the Western Pacific region (n=20, 19.4%). Very few posts contained a health-related disclaimer (n=7, 6.8%), and 18.4% (n=19) of posts mentioned COVID-19.

**Table 1:** Characteristics of Instagram posts about hepatitis B (N=103).

Category			Posts, n (%)
**Graphics**			
	Image only	31 (30.1)	
	Text only	10 (9.7)	
	Mix text and image	38 (36.9)	
	Video	8 (7.8)	
	Infographic	16 (15.5)	
**Creator expertise**			
	Health expertise	38 (36.9)	
	No health expertise	65 (63.1)	
**Creator status**			
	For profit	38 (36.9)	
	Not for-profit	65 (63.1)	
**Audience target^a^**			
	Prevention	21 (20.4)	
	Patients and caregivers	32 (31.1)	
	Health care providers	9 (8.7)	
	Other or not specified	52 (50.5)	
**Country or region**			
	Africa	16 (15.2)	
	Americas	40 (38.8)	
	Southeast Asia	15 (14.6)	
	European	7 (6.8)	
	Eastern Mediterranean	5 (4.9)	
	Western Pacific	20 (19.4)	
**Disclaimers**			
	Yes	7 (6.8)	
	No	96 (93.2)	
**Mentions COVID-19^b^**			
	Yes	19 (18.6)	
	No	84 (81.5)	

^a^Multiple audiences could be selected to percentages to not equal 100%.

^b^Percentages shown among those posts promoting a product or service.

### Characteristics of Instagram Profiles That Posted About HBV

A minority group of content creators claimed to have some kind of health expertise either in the post or on their Instagram profile (38/103, 36.9%). Similarly, a minority group of content creators claimed to be for-profit (38/103, 36.9%), meaning the majority claimed to be from not-for-profit sources (63.1%).

### Prevalence of and Engagement With HBV Claims and Misinformation on Instagram

The most common types of claims contained some kind of activism related to HBV (50/103, 48.5%; Table 2). These posts often included awareness of HBV causes and treatments, and there was no difference in misinformation prevalence among posts with claims about activism. However, for all the remaining claims, posts about treatment (55.6% vs 44.4%; *χ*²_1_=12.7; *P*<.001), cures (75% vs 25%; Fisher *P*<.001), natural remedies (92.3% vs 7.7%; Fisher *P*<.001), symptoms (60% vs 40%; *χ*²_1_=13.2; *P*<.001), and censorship (66.7% vs 33.3%; Fisher *P*=.005) contained significantly higher proportions of misinformation than accurate information. In contrast, there were significantly lower proportions of misinformation in posts about prevention (7.4% vs 92.6%; exact*, P*=.032) and other comments (39.4% vs 60.6%; *χ*²_1_=7.0; *P*=.008) than accurate information.

**Table 2:** Comparison of different types of claims by misinformation in posts about HBV on Instagram (N=103).

Claims category			Total (N=103), n (%)		Posts with each specific type of claims, n (%)		Posts without each specific type of claims, n (%)		*P* value^a^
**Activism**			50 (48.5)						.762
	Misinformation			11 (22)		13 (24.5)			
	No misinformation			39 (78)		40 (75.5)			
**Prevention**			27 (26.2)						*.032^b^*
	Misinformation			2 (7.4)		22 (28.9)			
	No misinformation			25 (92.6)		54 (71)			
**Treatment**			18 (17.5)						*<.001*
	Misinformation			10 (55.6)		14 (16.5)			
	No misinformation			8 (44.4)		71 (83.5)			
**Cure**			12 (11.6)						*<.001*
	Misinformation			9 (75)		15 (16.5)			
	No misinformation			3 (25)		76 (83.5)			
**Natural remedies**			13 (12.6)						*<.001*
	Misinformation			12 (92.3)		12 (13.3)			
	No misinformation			1 (7.7)		78 (86.7)			
**Symptoms**			15 (14.6)						*<.001*
	Misinformation			9 (60)		15 (17)			
	No misinformation			6 (40)		73 (83)			
**Anecdote**			10 (9.7)						*.036*
	Misinformation			5 (50)		19 (20.4)			
	No misinformation			5 (50)		74 (79.6)			
**Censorship conspiracies**			9 (8.7)						*.005*
	Misinformation			6 (66.7)		18 (19.1)			
	No misinformation			3 (33.3)		76 (80.9)			
**Other**			33 (32)						*.008*
	Misinformation			13 (39.4)		11 (15.7)			
	No misinformation			20 (60.6)		59 (84.3)			

^a^Fisher exact tests for variables with cells containing fewer than n=5; all other comparisons made with chi-square tests.

^b^Italics denotes statistical significance at *P*<.05.

Nearly a quarter of posts (24/103, 23.3%) contained misinformation about hepatitis B and hepatitis B treatment. Posts that contained HBV misinformation had higher engagement in the form of likes overall (1459.2 vs 941.8 engagements; *z*=−517.4; *P*<.001; Table 3). Content creators who shared misinformation about HBV had significantly fewer followers (24,075.7 vs 68,192.2 followers; *z*=44,116.5; *P*<.001) but were following significantly more accounts (1322.8 vs 910.7 accounts; *z*=−412.1; *P*<.001) than those who shared accurate information about HBV.

**Table 3:** Comparison of engagement with posts on Instagram that contain misinformation about HBV (N=103).

Claims category	Misinformation (n=24)			No misinformation (n=79)			*P* value^a^
	Mean (SD)	95% CI	Mean (SD)		95% CI		
Likes	1459.2 (1)	1458.8-1459.6	941.8 (1)		941.6-942.0	<.001	
Followers	24,075.7 (1)	24,075.3-24,076.1	68,192.2 (1)		68,192.0-68,192.4	<.001	
Following	1322.8 (1)	1322.4-13,232	910.7 (1)		910.4-910.9	<.001	

^a^*z* tests for comparing means.

### Association of HBV Claims With Misinformation by Validity Indicators on Instagram

Validity indicators included variables representing the use of academic or government citations (yes or no), personal anecdotal experiences (yes or no), and the use of disclaimers to contextualize claims about hepatitis B (yes or no). When examined by misinformation status only, the use of anecdotes was marginally significantly different; 20.4% (19/103) of posts with anecdotes used misinformation compared to 79.6% of posts with anecdotes having accurate information (Fisher *P*=.036; Table 2).

### Profitability of HBV Claims and Misinformation on Instagram

Significantly more misinformation posts were for-profit (39.5% vs 13.8%; *χ*²_1_=8.8; *P*=.003) compared to accurate posts (Table 4). Among posts with misinformation, 39.5% (15/24) were for-profit. The majority of censorship conspiracies, which were primarily about governments intentionally infecting and censoring citizens with HBV, were not profitable, 88.9% (8/9, Table 4). For-profit accounts were following a significantly higher number of accounts than those that were not for-profit (b=750.3, 95% CI 148.8-1351.8; *P*=.015; data not shown).

**Table 4:** Comparison of misinformation and censorship conspiracies by misinformation in posts about HBV on Instagram (N=103).

		Profitability	No profitability	*P* value^a^	
**Misinformation content**					*.003^b^*
	Misinformation	15 (39.5)	9 (13.8)		
	No misinformation	23 (60.5)	56 (86.1)		
**Censorship conspiracies**					.284
	Yes	1 (11.1)	8 (88.9)		
	No	29 (30.9)	65 (69.1)		

^a^Fisher exact tests for variables with cells containing fewer than n=5; all other comparisons made with chi-square tests.

^b^Italics denotes statistical significance at *P*<.05.

## Discussion

### Principal Findings

The COVID-19 pandemic heightened public awareness of online misinformation, garnering the US Surgeon General’s advisory in 2021 [41]. Since then, there has been a proliferation in research on the health impacts of social media use and how misinformation may influence patient health behaviors and health outcomes. Although medical misinformation is not a new health care issue, the internet has facilitated its more virulent dissemination [8]. Our results suggest this is the case for HBV misinformation on social media. Concerningly, 1 of 4 posts we sampled about HBV contained misinformation, and more than one-third of these posts were for monetary gain. A greater proportion of the accounts with misinformative posts were for profit. The typical motives are monetary gain, posing natural cures and false treatment options as opposed to promoting a patient's health interest. There is also a distinct lack of disclosures associated with profitability, which may bias information consumption among consumers. Disclosures are important indicators of potential bias that influence consumer information consumption. This formative research reveals the prevalence and types of misinformed claims about HBV on Instagram.

A notable finding in our formative study is the differential geographic distribution of content creators that shared HBV misinformation. While most posts were made by accounts originating in the Americas, most misinformation posts came from the African continent, suggesting that HBV misinformation differs by global region and may further disparities in information availability. Information inequality is the idea that individuals from impoverished backgrounds (eg, less educated and lower health literacy and incomes) access fewer information resources and thus experience unequal access to high-quality information [42]. Information inequality in action may predispose individuals from low socioeconomic backgrounds to pernicious and misleading online health misinformation. While the differential regional impact of online HBV misinformation was beyond the scope of this study, we recommend this as a priority for future research in this area, given the association of health literacy with poorer health outcomes in more rural and remote areas [43,44].

These findings suggest potential clinical relevance for patient education, online health information seeking, and shared decision-making in the management of acute and chronic HBV. For patients who experience symptomatic or chronic HBV, there are regimens to ameliorate or treat lifelong disease [45]. However, the efficacy and availability of these regimens may be unknown to patients. Online information about HBV is unregulated, and nearly a quarter (24/103, 23%) of the posts in our study had misinformation, although this was only a snapshot in time and cannot be generalized to the prevalence of HBV on Instagram over time. Many misinformed posts contained conspiracy theories (30%) on Instagram. Social media disseminated misinformation is a potential barrier to prevention or initiation and sustained management of HBV that deserves ongoing attention in clinical and research agendas.

The majority of HBV information we evaluated on Instagram did not contain misinformation, and these accounts had significantly more followers than accounts proliferating misinformation. However, accounts that spread misinformation had higher engagement and followed more accounts than accounts with accurate HBV information. Overall, the virality and reception of misinformation in our sample of HBV posts outweighed that of accurate posts. While we did not evaluate health outcomes in this study, the most concerning *potential* effects of misinformed Instagram posts about HBV are with potential harm from treatment avoidance, adverse effects, or medication interaction of HBV-related products. With 30% of Instagram posts that had misinformation containing conspiracy theories, medication nonadherence, missed follow-up appointments, and progression of chronic hepatitis B to cirrhosis are of potential concern. Misinformation has the potential to contribute to adverse health outcomes and it can also sow seeds of distrust in health institutions and health care professionals [46].

Formative methodological approaches to describing online HBV misinformation can extend to other gastrointestinal pathologies in future research. This study provides an initial overview of HBV misinformation on Instagram, providing a foundation for future work. Qualitative research examining the narratives, themes, and user interactions surrounding this content could yield deeper insight into how HBV misinformation is framed and spread online. Additional research on the impact of social media in HBV care can improve clinicians’ readiness to respond effectively to patient concerns related to online misinformation. By documenting the initial prevalence of and engagement with online misinformation about HBV, we lay the groundwork for monitoring the potential differential and unequal impact of HBV misinformation among subpopulations of individuals with HBV. Still, barriers to conducting research on misinformation remain, such as limited funding opportunities [47].

### Limitations

Our study is limited by the cross-sectional nature of data collection, small sample, and the inability to tie HBV misinformation directly to patient health outcomes. However, due to the need for contextual and descriptive analysis of HBV topics to inform hypothesis generation and the development of larger more complex intervention trials [39,40], this research is critically needed as we are aware of no other prior studies that have contextualized the topics of popular HBV content on Instagram. Further research applying more sophisticated classification tools on larger, more robust samples of social media posts about HBV would provide more generalizable estimates of HBV misinformation prevalence. This future research is needed to fully understand and address the downstream effects of HBV misinformation on health behaviors, health decision-making, and, ultimately, health outcomes. Given the dynamic nature of social media, our findings may not be generalizable to other periods. To enhance transferability and relevance of the findings, we conducted a small replication study in September 2025 (n=60 posts, 30 from each search term), which exhibited similar topics of misinformation and a nearly identical proportion of misinformation (33% of posts, compared to 30% in the original sample from December 2021). Additional engagement measures, such as aggregated comments or shares, were not available at the time of data collection, which limits a more detailed evaluation of user engagement.

### Conclusions

Social media are increasingly used by patients to seek health information; however, the quality of HBV information online appears to be inconsistent based on our sample. In this formative study, HBV misinformation had more engagement than accurate information on Instagram, and HBV misinformation was more likely to be for-profit than accurate information. This suggests that HBV misinformation spreads more easily than accurate information, suggesting that people searching for HBV information on Instagram may be targeted with false, profit-driven claims about treating, or curing HBV. In turn, misleading marketing may influence health decisions in harmful ways. The primary innovation of our findings lies in the characterization of visual social media information and misinformation about HBV on Instagram, a platform that has been understudied in health misinformation research, given the unique visual format of information. More research is needed to estimate the broader prevalence of HBV misinformation and its influence on health beliefs, behaviors, and outcomes. Improving media literacy may help reduce the influence of misleading HBV information online. The issues illustrated in this formative study do not solely affect the field of hepatology. Online medical misinformation is a longstanding challenge, exacerbated by recent technological advances, that requires clinicians and researchers alike to recognize a paradigm shift in how patients access information about HBV and other gastrointestinal diseases. Our study marks the first platform-specific investigation into HBV misinformation, suggesting that HBV misinformation is present on Instagram, particularly within the context of conspiracy theories and from accounts designed for monetary gain. This descriptive information can inform hypothesis generation, future classification of more robust social media samples of HBV content, and future health communication interventions [39,40].

## Data Availability

The datasets generated or analyzed during this study are available from the corresponding author on reasonable request.

## Appendix

Multimedia Appendix 1 STROBE checklist.

## Abbreviations

HBV: hepatitis B virus
STROBE: Strengthening the Reporting of Observational Studies in Epidemiology
OnC-M: Online Cancer Nutrition Misinformation
